# Supplementary material for: When Conventional Methods Fail: First Detection of a Candida viswanathii Outbreak in Europe in a Pediatric Hospital Revealed by Whole Genome Sequencing and FT-IR Spectroscopy
Source: Microorganisms. 2025 Nov 26;13(12):2698. doi: 10.3390/microorganisms13122698 (PMC12734905; doi:10.3390/microorganisms13122698)
Supplement: Supplementary file 1 [file microorganisms-13-02698-s001.zip › Figure S1.pdf]

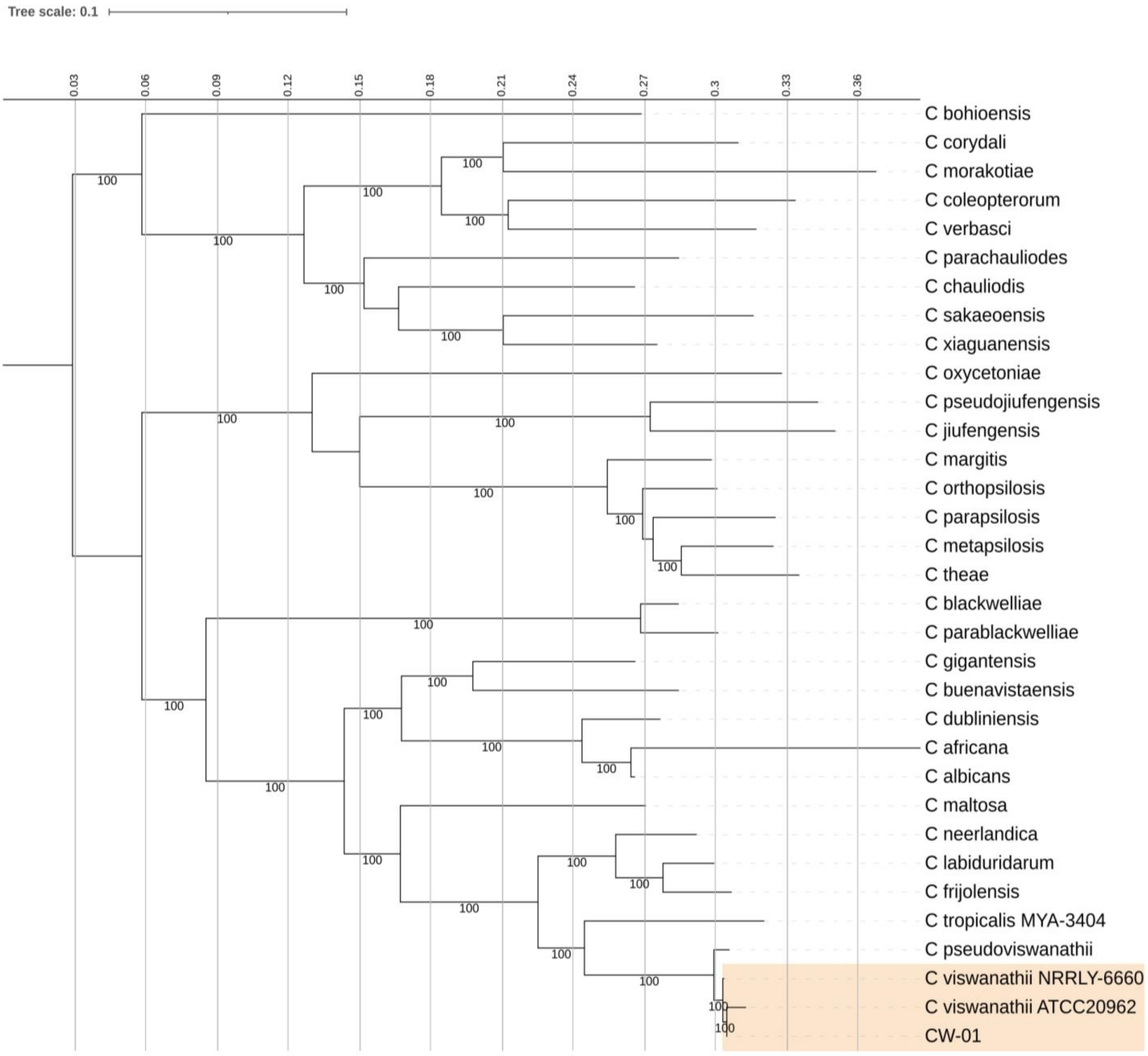

Figure S1. Estimated Maximum Likelihood analysis from concatenated fungal marker genes extracted using the UFCG pipeline from clinical sample (n=1) and genomes from Candida genus (n=32). The tree was inferred from concatenated fungal marker genes extracted using the UFCG pipeline. The phylogeny was estimated with IQTREE using the best-fit model of nucleotide substitution Q.insect+F+I+R4 with 1,000 replicates and fast bootstrapping. Bootstrap values higher than 90 are shown on branches. The tree was edited in iTOL (v6.9).
