# Supplementary material for: When Conventional Methods Fail: First Detection of a Candida viswanathii Outbreak in Europe in a Pediatric Hospital Revealed by Whole Genome Sequencing and FT-IR Spectroscopy
Source: Microorganisms. 2025 Nov 26;13(12):2698. doi: 10.3390/microorganisms13122698 (PMC12734905; doi:10.3390/microorganisms13122698)
Supplement: Supplementary file 1 [file microorganisms-13-02698-s001.zip › Figure S2.pdf]

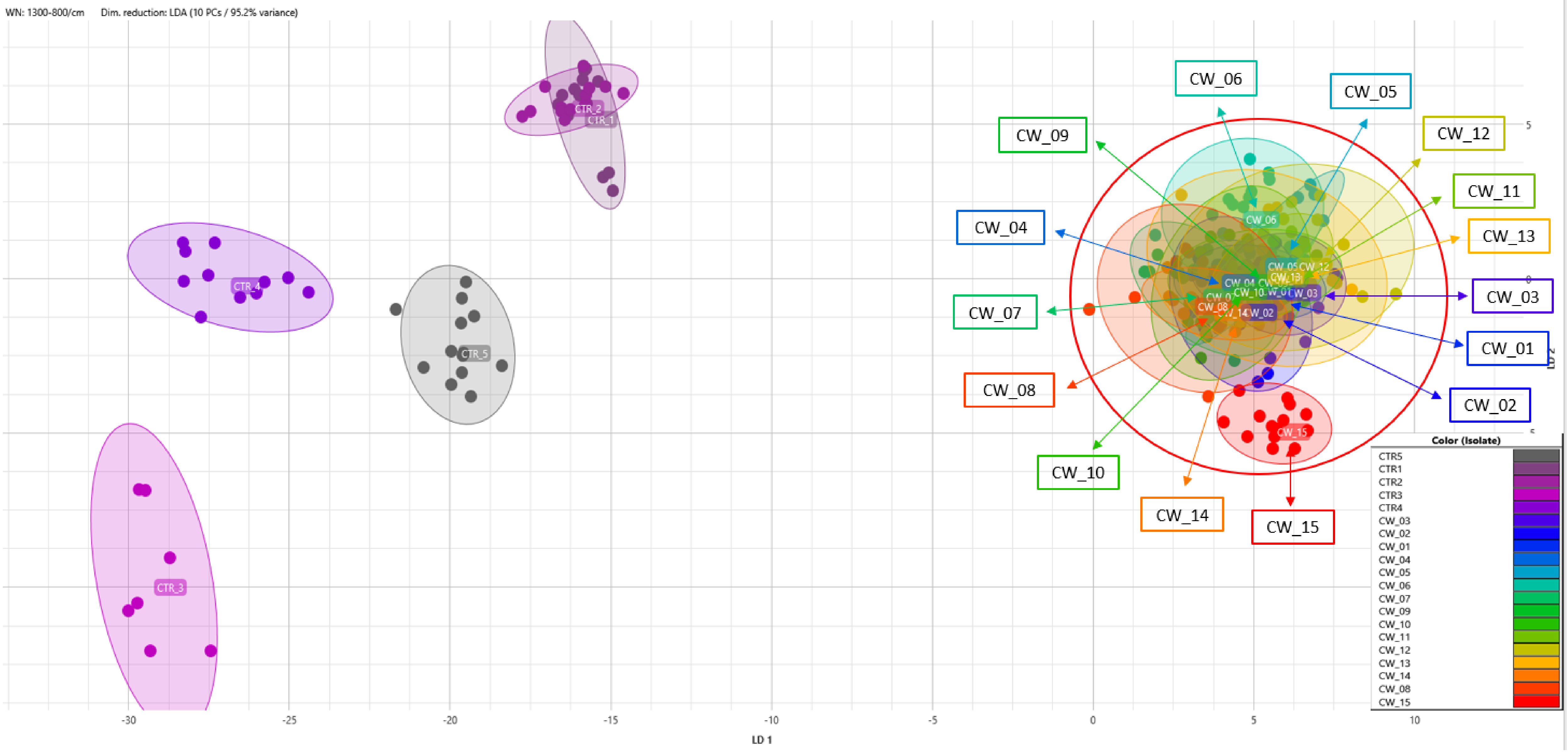

Figure S2. Scatter plot generated by FT-IR Bruker analysis (spectral range 1300–800 cm^{-1}; dimensionality reduction by LDA using 9 principal components, explaining 95.7% of the variance) showing the *Candida viswanathii* outbreak isolates clustering together. Four external *Candida* strains, used as controls, are clearly separated into distinct groups. Ellipses represent 95% confidence intervals for each cluster.
