# Supplementary material for: When Conventional Methods Fail: First Detection of a Candida viswanathii Outbreak in Europe in a Pediatric Hospital Revealed by Whole Genome Sequencing and FT-IR Spectroscopy
Source: Microorganisms. 2025 Nov 26;13(12):2698. doi: 10.3390/microorganisms13122698 (PMC12734905; doi:10.3390/microorganisms13122698)
Supplement: Supplementary file 1 [file microorganisms-13-02698-s001.zip › Figure S3.pdf]

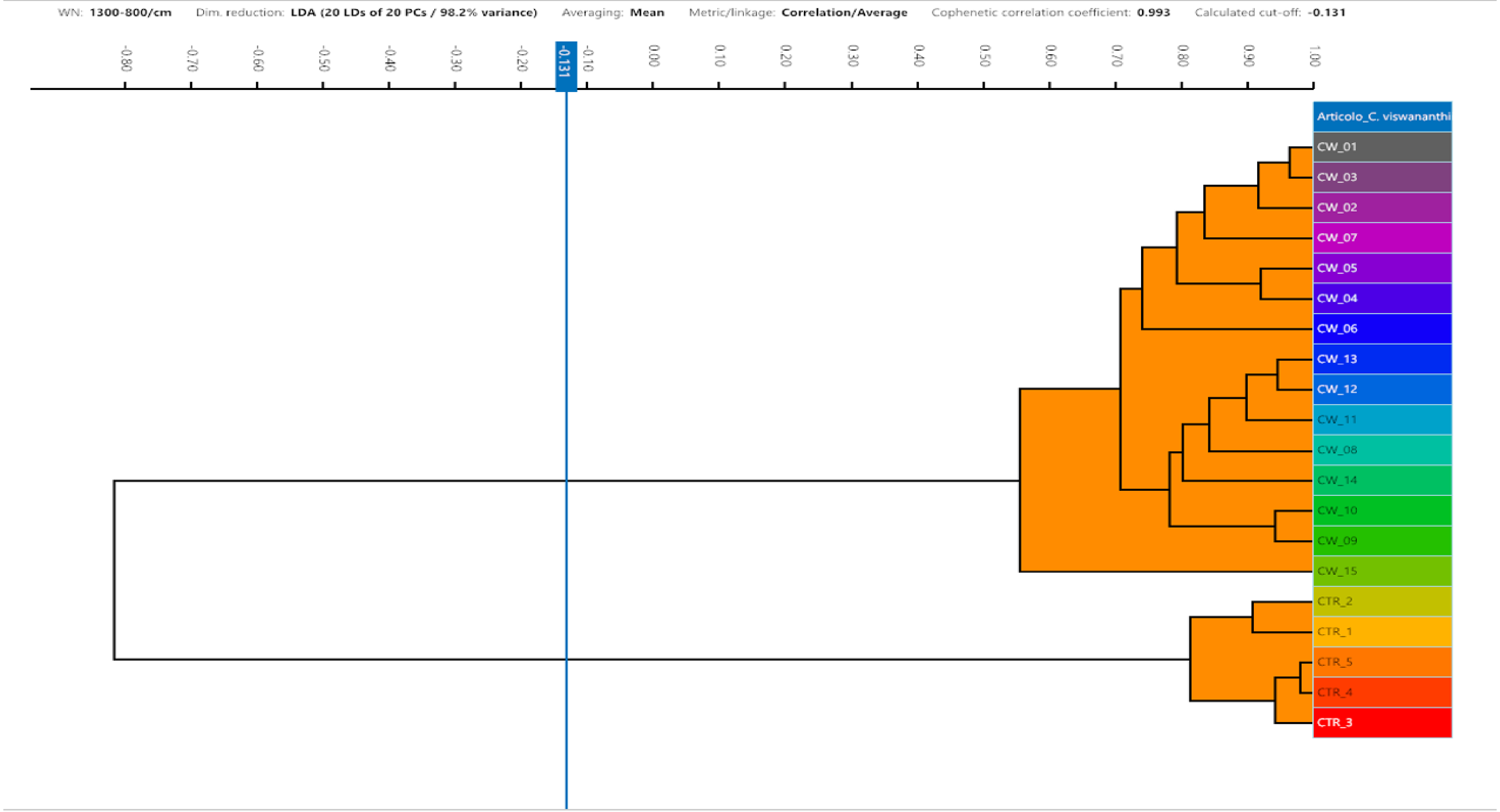

Figure S3. Dendrogram generated by FT-IR Bruker analysis (spectral range 1300–800 cm^{-1}; dimensionality reduction by LDA using 18 linear discriminants from 20 principal components, explaining 98.7% of the variance) showing the clustering of *Candida viswanathii* outbreak isolates together. Four external *Candida* strains, used as controls, are clearly separated from the outbreak cluster. The analysis was performed using the Euclidean distance metric and average linkage, with a cophenetic correlation coefficient of 0.933 and a calculated cut-off value of 29.51.
