# Supplementary material for: When Conventional Methods Fail: First Detection of a Candida viswanathii Outbreak in Europe in a Pediatric Hospital Revealed by Whole Genome Sequencing and FT-IR Spectroscopy
Source: Microorganisms. 2025 Nov 26;13(12):2698. doi: 10.3390/microorganisms13122698 (PMC12734905; doi:10.3390/microorganisms13122698)
Supplement: Supplementary file 1 [file microorganisms-13-02698-s001.zip › Figure S4.pdf]

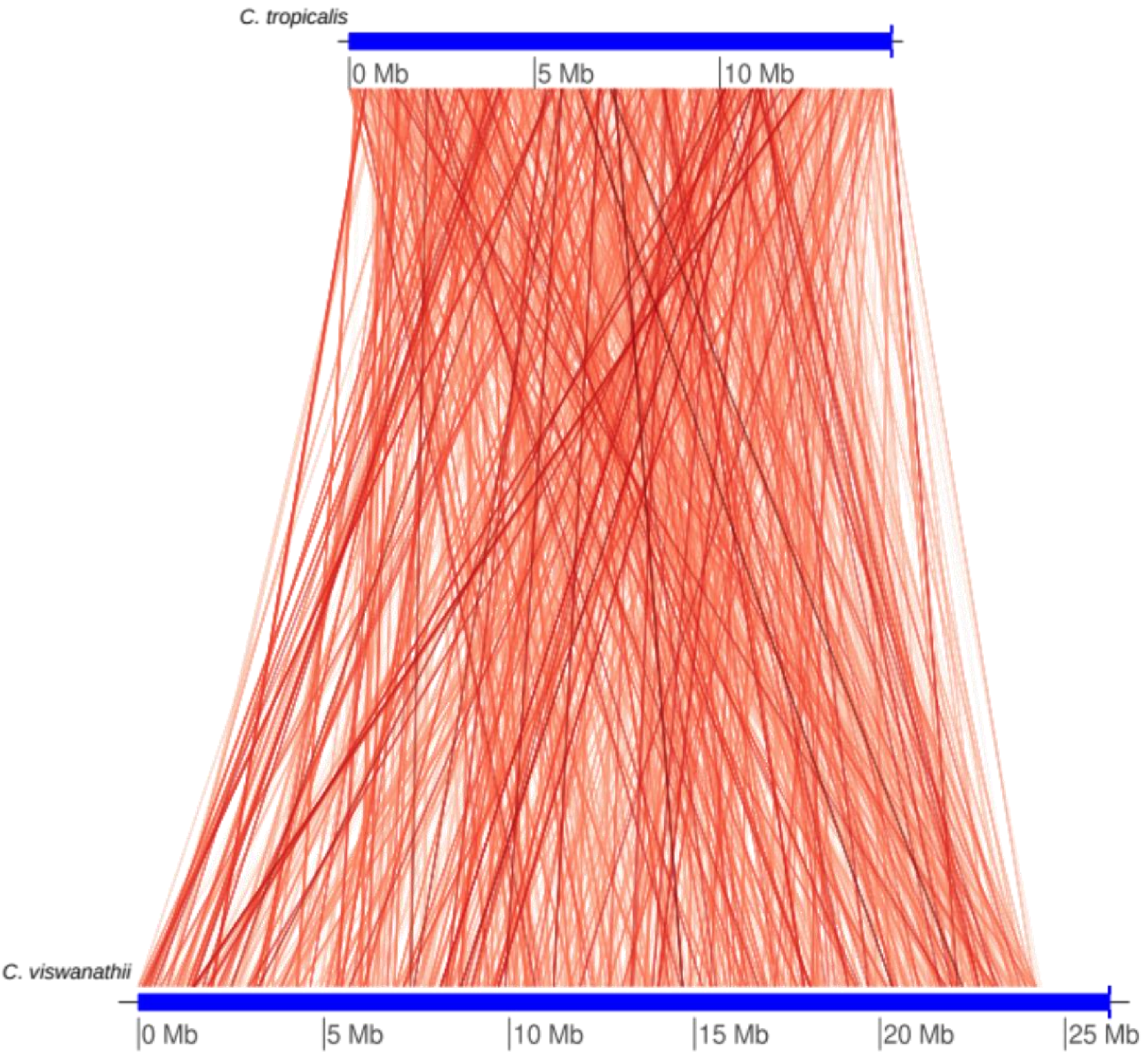


**Figure S4. Visualization of conserved region between *C. tropicalis* and *C. viswanathii* (sample CW_01) genomes by fastANI**
