# Supplementary material for: When Conventional Methods Fail: First Detection of a Candida viswanathii Outbreak in Europe in a Pediatric Hospital Revealed by Whole Genome Sequencing and FT-IR Spectroscopy
Source: Microorganisms. 2025 Nov 26;13(12):2698. doi: 10.3390/microorganisms13122698 (PMC12734905; doi:10.3390/microorganisms13122698)
Supplement: Supplementary file 1 [file microorganisms-13-02698-s001.zip › Figure S5.pdf]

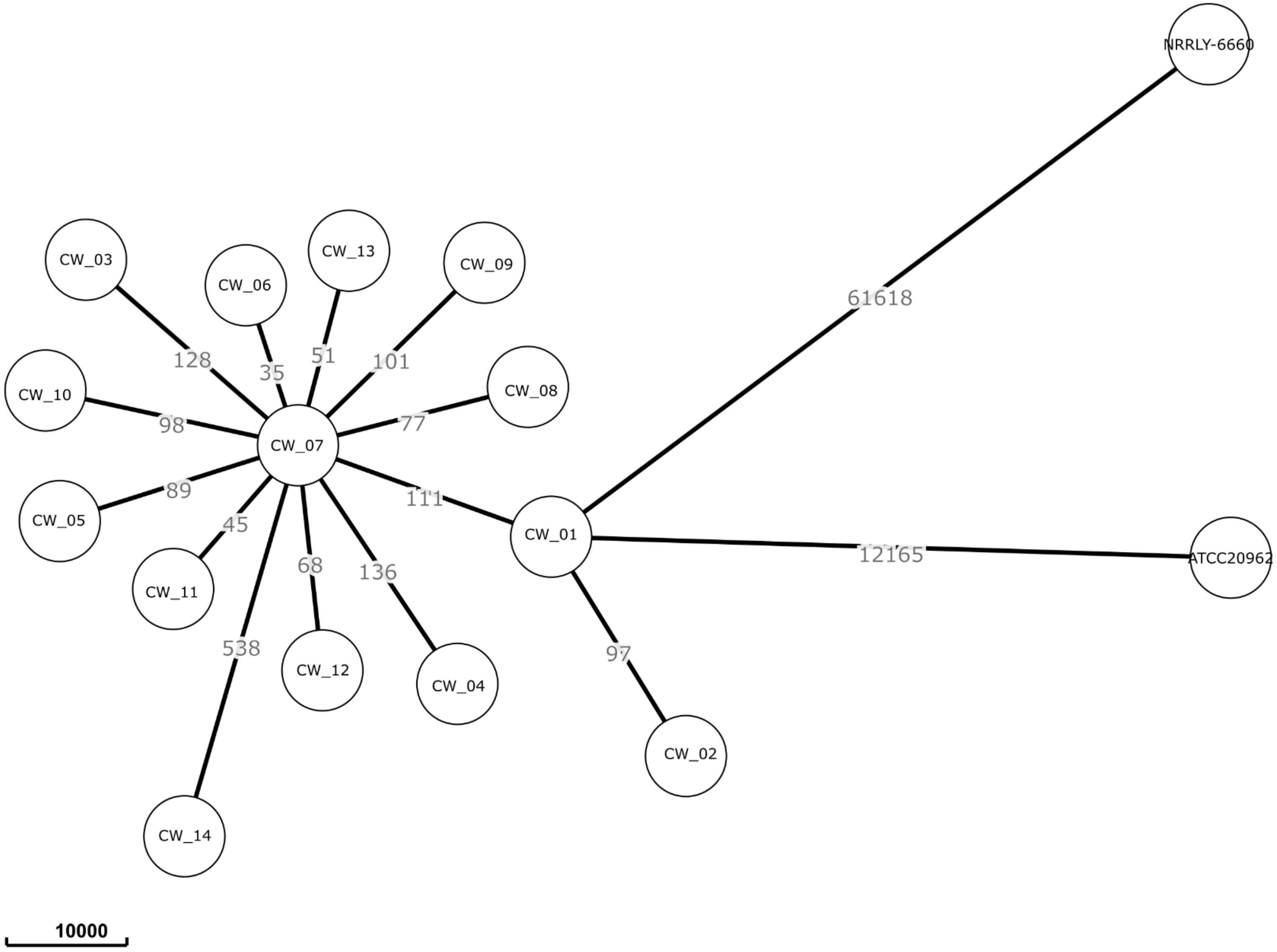

Figure S5. *Minimum spanning tree (WGS-SNP analysis) of Candida viswanathii outbreak isolates.*
All Italian isolates formed a highly homogeneous cluster, consistent with a single clonal lineage. Reference strains NRRL Y-6669 and ATCC 20963 were separated by >10,000 SNPs, indicating marked genomic divergence and supporting the emergence of a distinct lineage.
